# A Prognostic Signature Constructed by *CTHRC1* and *LRFN4* in Stomach Adenocarcinoma

**DOI:** 10.3389/fgene.2021.646818

**Published:** 2021-08-26

**Authors:** Songling Han, Wei Zhu, Weili Yang, Qijie Guan, Chao Chen, Qiang He, Zhuoheng Zhong, Ruoke Zhao, Hangming Xiong, Haote Han, Yaohan Li, Zijian Sun, Xingjiang Hu, Jingkui Tian

**Affiliations:** ^1^The Cancer Hospital of the University of Chinese Academy of Sciences (Zhejiang Cancer Hospital), Institute of Basic Medicine and Cancer (IBMC), Chinese Academy of Sciences, Hangzhou, China; ^2^College of Biomedical Engineering and Instrument Science, Zhejiang University, Hangzhou, China; ^3^Changshu Qiushi Technology Co., Ltd., Suzhou, China; ^4^Department of Gastrointestinal Surgery, The First Affiliated Hospital, Zhejiang University School of Medicine, Hangzhou, China; ^5^Department of Nephrology, Zhejiang Provincial People’s Hospital, Hangzhou, China; ^6^Zhejiang Provincial Key Laboratory for Drug Evaluation and Clinical Research, The First Affiliated Hospital, Zhejiang University School of Medicine, Hangzhou, China

**Keywords:** stomach adenocarcinoma, bioinformatics analysis, differentially expressed genes, biomarker, Gene Expression Omnibus

## Abstract

**Background:**

Stomach adenocarcinoma (STAD) is the most common histological type of stomach cancer, which causes a considerable number of deaths worldwide. This study aimed to identify its potential biomarkers with the notion of revealing the underlying molecular mechanisms.

**Methods:**

Gene expression profile microarray data were downloaded from the Gene Expression Omnibus (GEO) database. The “limma” R package was used to screen the differentially expressed genes (DEGs) between STAD and matched normal tissues. The Database for Annotation, Visualization, and Integrated Discovery (DAVID) was used for function enrichment analyses of DEGs. The STAD dataset from The Cancer Genome Atlas (TCGA) database was used to identify a prognostic gene signature, which was verified in another STAD dataset from the GEO database. CIBERSORT algorithm was used to characterize the 22 human immune cell compositions. The expression of *LRFN4* and *CTHRC1* in tissues was determined by quantitative real-time PCR from the patients recruited to the present study.

**Results:**

Three public datasets including 90 STAD patients and 43 healthy controls were analyzed, from which 44 genes were differentially expressed in all three datasets. These genes were implicated in biological processes including cell adhesion, wound healing, and extracellular matrix organization. Five out of 44 genes showed significant survival differences. Among them, *CTHRC1* and *LRFN4* were selected for construction of prognostic signature by univariate Cox regression and stepwise multivariate Cox regression in the TCGA-STAD dataset. The fidelity of the signature was evaluated in another independent dataset and showed a good classification effect. The infiltration levels of multiple immune cells between high-risk and low-risk groups had significant differences, as well as two immune checkpoints. *TIM-3* and *PD-L2* were highly correlated with the risk score. Multiple signaling pathways differed between the two groups of patients. At the same time, the expression level of *LRFN4* and *CTHRC1* in tissues analyzed by quantitative real-time PCR were consistent with the *in silico* findings.

**Conclusion:**

The present study constructed the prognostic signature by expression of *CTHRC1* and *LRFN4* for the first time *via* comprehensive bioinformatics analysis, which provided the potential therapeutic targets of STAD for clinical treatment.

## Introduction

Stomach adenocarcinoma (STAD), the most common histological type (∼95%) of malignancy originating in the stomach, imposed a considerable global health burden (Ajani et al., 2017). So far, there is no sensitive and specific diagnostic marker for early diagnosis of STAD (Duffy et al., 2014). Although several drugs, such as trastuzumab, ramucirumab, and immune checkpoint inhibitors, had been used for the treatment of STAD in clinics, the survival rates of patients in advanced stages remained low (Bang et al., 2010; Fuchs et al., 2014, 2018). Therefore, it is urgent to identify novel prognostic biomarkers for STAD.

Over the past decades, the high-throughput sequencing generated large-scale biological data, and it has been an effective tool for discovering promising biomarkers for cancer (Jiang and Liu, 2015). Many biomarkers such as *AFP*, *EGFR*, and *HER2* were discovered through bioinformatic analysis (Tateishi et al., 2008; Vizoso et al., 2015; Alix-Panabières and Pantel, 2016). Several predictive signatures of gene expression had great significance in clinical prognosis applications as well as biomarker identification. For example, Long et al. (2018) constructed a prognostic signature for patients with hepatocellular carcinoma based on RNA sequencing data. Furthermore, a five-gene signature-derived risk score module based on data from The Cancer Genome Atlas (TCGA) databases accurately predicted gastric cancer (GC) prognosis (Zhao et al., 2019). Identification of genes that significantly correlated with progression in STAD patients might be applicable for establishing robust module as well, which could provide prognosis for STAD treatment; however, it has not been investigated.

In the present study, we analyzed the mRNA expression profiles of STAD from the Gene Expression Omnibus (GEO) database for screening of the differentially expressed genes (DEGs). Then, gene ontology (GO) analysis was conducted to reveal the main biological functions modulated by these DEGs. Through the correlation analysis of patients’ survival information with STAD from TCGA, the candidate genes closely related to the survival rate of patients were identified. Cox analysis was performed for evaluating mRNAs correlation with survival rates and construction of a two-gene signature that indicates the relationship to immune system responses. In addition, another independent dataset was analyzed to verify the prognostic effect of the signature. The results indicated that the high-risk group was associated with tumor-associated pathways based on the two-gene signature derived risk score signature. The expression of these two genes was further verified in the recruited patients using quantitative real-time polymerase chain reaction (qRT-PCR).

## Materials and Methods

### Data Collection and Screening of DEGs

Gene expression profile microarray data (GSE118916 of 15 STAD and 15 healthy tissues, GSE13861 of 65 STAD and 19 healthy tissues, and GSE103236 of 10 STAD and 9 healthy tissues) were downloaded from the GEO database.^1^ The “limma” R package was used to screen the DEGs between STAD and matched normal tissues (Ritchie et al., 2015). Adjusted *p*-value < 0.05 and | log2 fold change (FC)| > 1 were set as the thresholds for DEGs identification. *p*-value adjustment used the built-in correction method of limma package.

### GO Enrichment Analyses

The Database for Annotation, Visualization, and Integrated Discovery (DAVID)^2^ was used for biological process (BP), cellular component (CC), and molecular function (MF) enrichment analyses of DEGs (Huang et al., 2007). *p*-value < 0.05 was used to screen statistically significant terms. The background gene set used the intersection of the gene expression lists of GSE118916, GSE13861, and GSE103236 to replace the default gene set.

### TCGA Data Analysis and Survival Analysis of DEGs

The data of STAD cases with both RNA sequencing and clinical information of TCGA were obtained from the Genomic Data Commons (GDC) data portal.^3^ The information on molecular subtype was obtained from previous studies (Adam et al., 2014; Rooney et al., 2015; Hause et al., 2016; Davoli et al., 2017). Cases with missing clinical information were deleted and 370 cases were retained. The mRNA high-level and low-level grouping was based on the median expression value of the mRNA. Survival curves were analyzed by the Kaplan–Meier method (Guyot et al., 2012). *p*-value < 0.05 was considered as statistically significant data.

### Construction and Validation of Prognostic Signature

We used R 3.6.2 with “survival” package to univariate Cox regression analysis and multivariate Cox regression. To reduce the number of mRNAs with similar expressions, mRNAs with *p*-value < 0.05 of univariate Cox regression were subjected to a stepwise multivariate Cox regression for construction of the prognostic signature. This signature was used to evaluate the survival prognosis of patients in TCGA-STAD datasets using a Kaplan–Meier curve, and log-rank test according to median value grouping of risk score. Reliability of the prognostic signature was assessed using the area under the curve (AUC) of the receiver operating characteristic (ROC) curve. Gene expression profile microarray data (GSE15459 of 200 gastric cancer cases; only 192 patients have clinical information) were downloaded from the GEO database to validate the reliability and prognostic value of two-gene signature using the ROC and Kaplan–Meier curves.

### Assessment of Immune Infiltration

We used the CIBERSORTx algorithm and the LM22 gene signature, which is a widely used approach to characterize the 22 human immune cell composition, including B cells, T cells, natural killer cells, and macrophages (Newman et al., 2019). After uploading the gene expression data with standard annotation on the CIBERSORTx web portal,^4^ the algorithm ran under LM22 signature and 1,000 permutations.

### Gene Set Enrichment Analysis

Gene set enrichment analysis (GSEA)^5^ was used to identify the promising signaling pathways for the high-risk group based on the risk score module (Subramanian et al., 2005). The file (c2.cp.kegg.v7.2.symbols.gmt) was selected for use as the reference gene file. False discovery rate (FDR) *q*-value < 0.05 and | normalized enrichment score (NES)| > 0.7 was chosen as the cutoff criterion. The gene set used the intersection of the default gene set and detected expressed genes.

### Patient Samples

A total of six STAD tissue samples and paired adjacent noncancerous tissue samples were obtained from the First Affiliated Hospital of Zhejiang University. The research has been carried out in accordance with the World Medical Association Declaration of Helsinki. It was approved by the Ethics Committee of The First Affiliated Hospital of Zhejiang University and the informed consent was obtained from all the recruited patients. All specimens had not been subjected to any preoperative radiotherapy or chemotherapy and were immediately soaked in RNA stabilization solution and stored at –80°C.

### qRT-PCR Analysis

Total RNA was extracted from six pairs of STAD tissues and adjacent normal tissues frozen in liquid nitrogen with TRIzol reagent (TIANGEN, Beijing, China). The extracted mRNA was diluted according to the concentration and then reverse-transcribed into complementary DNA using a reverse transcription kit (abm, Vancouver, Canada). qRT-PCR was performed on the LightCycler96 Sequence Detection System (BIO-RAD, California, United States) with BlastTaq^TM^ (abm). The thermocycling conditions were as follows: Initial denaturation at 95°C for 3 min, followed by 45 cycles of denaturation at 95°C for 15 s and annealing at 60°C for 1 min. The primer sequences for PCR amplification were as follows: *LRFN4*, forward: 5′-ACAACTTCATCCAGGCCCTG-3′, reverse: 5′-AGGATGAGGTGCTGCAGATT-3′; *CTHRC1*, forward: 5′-CTTGGGAAAATTGCGGAGTG-3′, reverse: 5′-TTC ATTTCAGGGCTTCCTTG-3′; *GAPDH*, forward: 5′-TGATGAG GAGAATTACTTGGAT-3′, reverse: 5′-CTTGGGATACTGCT TGACA-3′. The relative mRNA expression level was calculated by the 2^–ΔΔCt^ method taking *GAPDH* as the reference gene.

### Statistical Analysis

The Mann–Whitney test and the Student’s *t*-test were used for comparison between the groups, when two groups were compared. A threshold of *p* < 0.05 was considered statistically significant. The correlation between risk score and gene expression was evaluated by Pearson’s *R* and statistical significance. Gene expression data were processed by plus one and log2-transformed.

## Results

### Identification and Functional Enrichment of DEGs

**FIGURE 1:**
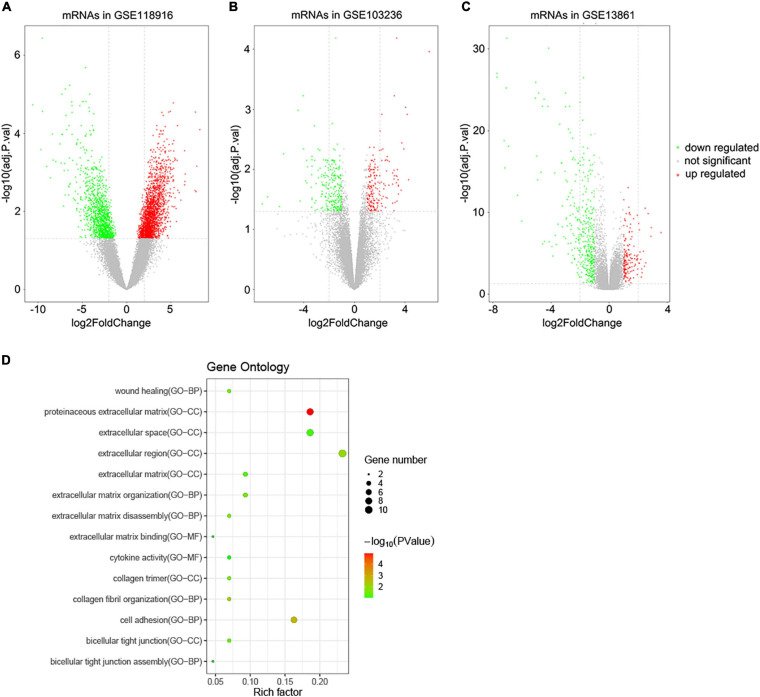
Volcano plots of DEGs in the three GEO datasets and functional enrichment of DEGs. **(A)** DEGs of the GSE118916 dataset. **(B)** DEGs of the GSE103036 dataset. **(C)** DEGs of the GSE13861 dataset. **(D)** Statistics of functional enrichment. CC represents cellular component, MF represents molecular function, and BP represents biological process.

A total of 3,860 DEGs were screened from the GSE118916 dataset by using the “limma” R package. In addition, 550 and 463 DEGs were screened from the GSE13861 and GSE103236 datasets, respectively. Volcano plots were plotted to present the distribution of DEGs between STAD and normal samples in each dataset (Figures 1A–C). Histograms were plotted to present the distribution of adjusted *p*-value in three datasets (Supplementary Figure 1). After the intersection, a total of 44 DEGs were identified as the intersection of the three datasets (Supplementary Table 1).

To reveal the biological functions of the 44 DEGs, GO enrichment analysis was conducted with DAVID. Regarding molecular function, the GO analysis results showed that the DEGs were mainly enriched in terms related to extracellular matrix binding and cytokine activity. These DEGs were involved in cell adhesion, wound healing, and extracellular matrix organization biological processes. For cellular components, the DEGs were enriched in extracellular regions, including the proteinaceous extracellular matrix, extracellular region, and extracellular space (Figure 1D).

### Survival Analysis of DEGs

**FIGURE 2:**
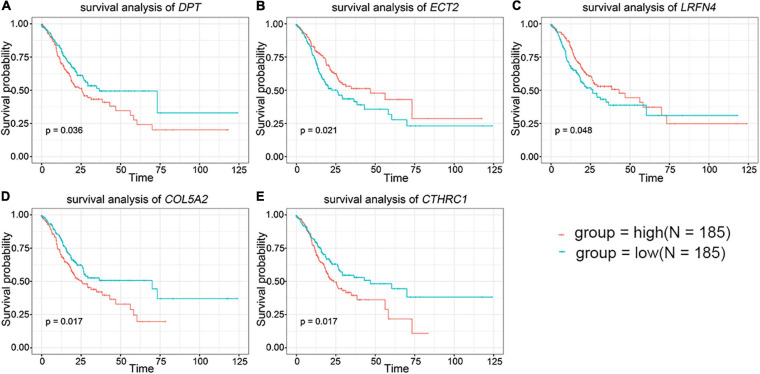
The prognostic value of key genes in the overall survival of stomach adenocarcinoma (STAD) patients. **(A)** *DPT.* **(B)** *ECT2*. **(C)** *LRFN4*. **(D)** *COL5A2*. **(E)** *CTHRC1*. The red lines signified individuals with high expression of gene and blue lines denoted those with low expression.

To further evaluate the prognostic value of the 44 DEGs, the clinical data of patients with STAD were downloaded from the TCGA database. The overall survival of patients with STAD based on the high and low expression of DEGs was then obtained using Kaplan–Meier plotters. The results indicated that the group of low-level expression of *DPT*, *COL5A2*, and *CTHRC1* and high-level expression of *ECT2* and *LRFN4* had a better survival in patients with STAD (Figure 2 and Supplementary Figure 2). In brief, five genes were significantly related to the prognosis of patients with STAD.

### Construction and Evaluation of a Two-Gene Signature

**FIGURE 3:**
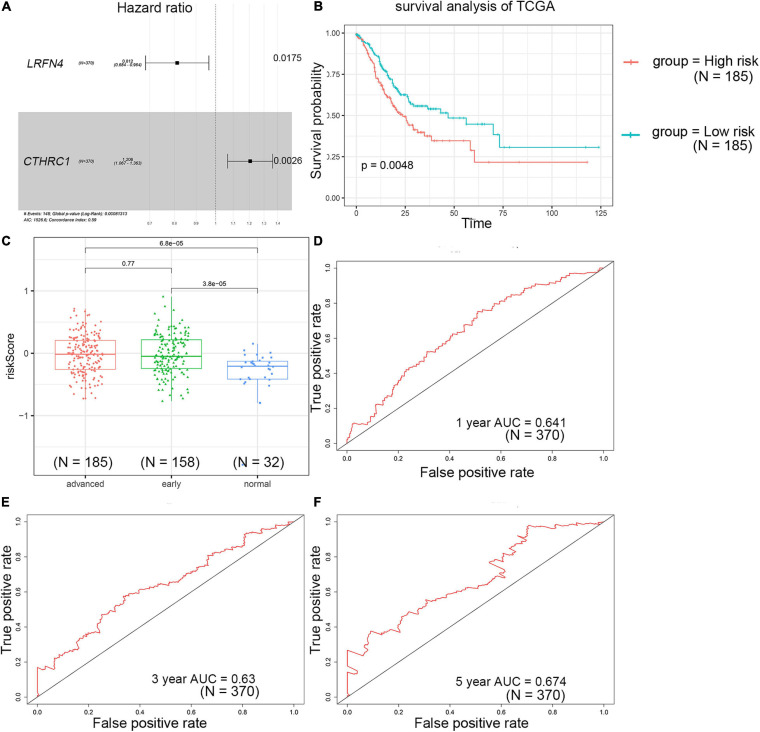
Prognostic value detection of *LRFN4* and *CTHRC1 via* multivariate Cox regression analysis in patients with STAD of TCGA dataset. **(A)** Multivariate Cox regression analysis of *LRFN4* and *CTHRC1.* **(B)** Survival analysis of the high-risk group and the low-risk group in the TCGA dataset. **(C)** Risk score in early stage and advanced stage of STAD in TCGA sets. **(D–F)** ROC curve of the prognostic signature in the TCGA dataset.

**FIGURE 4:**
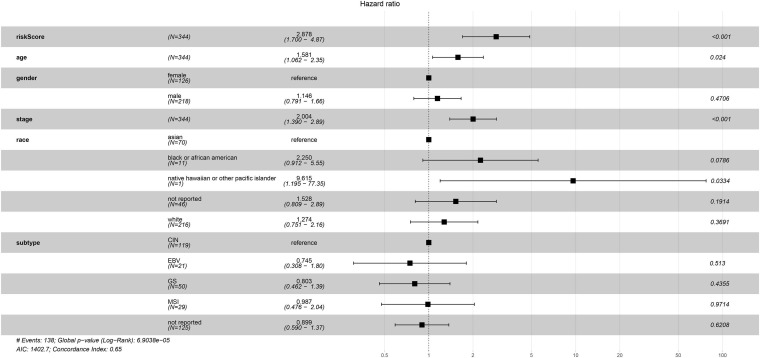
Multivariate Cox regression analysis of clinicopathologic factors and risk score for STAD in TCGA sets.

The univariate Cox regression analysis showed that five mRNAs were associated with overall survival in the TCGA-STAD dataset (*N* = 370). Three mRNAs with a *p*-value < 0.05 were selected for further analysis (Table 1). Two (*LRFN4* and *CTHRC1*) out of three mRNAs were screened out by stepwise multivariate Cox regression analysis (Figure 3A). Then, a two-gene signature was constructed by expression of *LRFN4* and *CTHRC1* and its coefficient in multivariate Cox regression as follows: Risk score = (–0.20788 × expression of *LRFN4*) + (0.18741 × expression of *CTHRC1*). According to the median value of risk scores, patients were divided into the high-risk group and the low-risk group; the high-risk group had a worse prognosis [(–0.77,0.91), median = –0.02; Figure 3B]. Remove the patients with missing age, sex, and tumor stage information and keep 344 samples for the next analysis. Risk score showed non-significant difference between early (I and II) and advanced (III and IV) stages in STAD patients, but it showed significant changes between early stage and normal (Figure 3C). The AUC-ROC of the signature for survival time was 0.641, 0.63, and 0.674 at 1, 3, and 5 years, respectively (Figures 3D–F). Multivariate Cox regression analyses also revealed that the risk score was an independent predictor of survival in TCGA datasets, after adjusting for age (<60 vs. ≥60), sex (male vs. female), tumor stage (I and II vs. III and IV), race, and molecular subtype (Figure 4).

**TABLE 1:** Prognostic value detection of the five genes in TCGA-STAD dataset.

Gene symbol	HR	HR.95L	HR.95H	*P*-value
*LRFN4*	0.825131	0.696212	0.977922	0.026587
*COL5A2*	1.231445	1.05985	1.430822	0.006544
*CTHRC1*	1.205681	1.065017	1.364924	0.003125
*ECT2*	0.900176	0.766669	1.056933	0.199163
*DPT*	1.09165	0.977869	1.21867	0.118414

*The hazard ratios (HRs) and 95% confidence intervals (CIs) were calculated by univariate Cox regression.*

### Validation of the Two-Gene Signature

**FIGURE 5:**
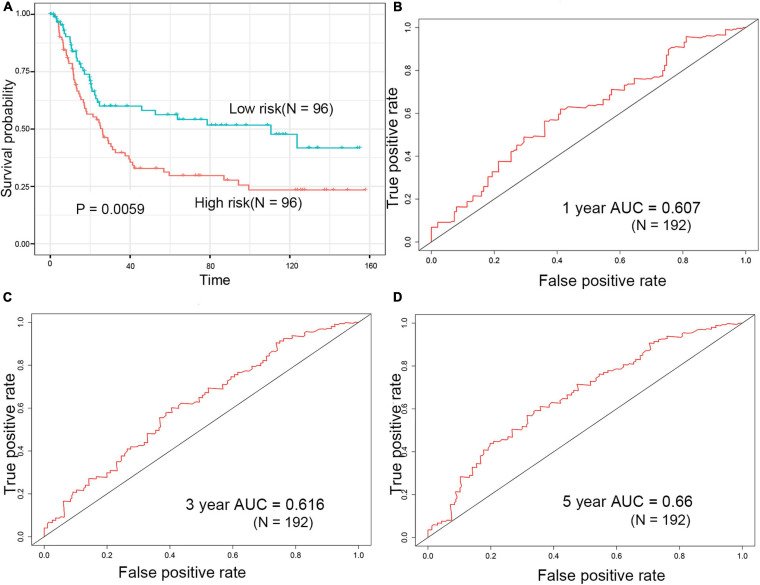
Evaluation of prognostic signature for over survival in the GEO dataset. **(A)** Survival analysis of the high-risk group and the low-risk group in the GEO dataset. **(B–D)** ROC curve of the prognostic signature in the GEO dataset.

GSE15459 dataset download from the GEO database (*N* = 200) was used to evaluate the repeatability and robustness of signature. The risk score of each patient was according to the same formula from the training dataset, and the median was used as the cutoff for patients categorized as low and high risk [(–0.29,1.38), median = 0.77]. Patients in the high-risk group had a significantly shorter survival time than those in the low-risk group (Figure 5A). The AUC-ROC of the signature for survival time was 0.607, 0.616, and 0.66 at 1, 3, and 5 years, respectively (Figures 5B–D).

### Comparison of the Two-Gene Signature With Other Signatures

Wu et al. (2020) constructed a nine-gene signature (include *CTHRC1*) that had a good prognostic capability for the survival of patients in the training dataset (TCGA-STAD dataset, *p*-value of KM curves = 2.80e–7) and validation dataset (GSE15459 dataset, *p*-value of KM curves = 0.011). Wang et al. (2016) constructed a 53-gene signature, the signature obtained achievement in the training dataset (TCGA-STAD dataset, *p*-value of KM curves = 1.41e–20) and validation dataset (GSE15459 dataset, *p*-value of KM curves = 0.02). In the present study, the two-gene signature showed a worse performance in the training dataset (*p* = 0.0048), but better performance in the validation dataset (*p* = 0.0059) compared to previous signatures. In addition, we performed an estimation using the same model and their model setting and the gene lists from those two articles. The performance of the 9-gene and 53-gene signature was also worse than the 2-gene signature (Supplementary Figure 3).

### Immune Landscape in Patients With STAD

**FIGURE 6:**
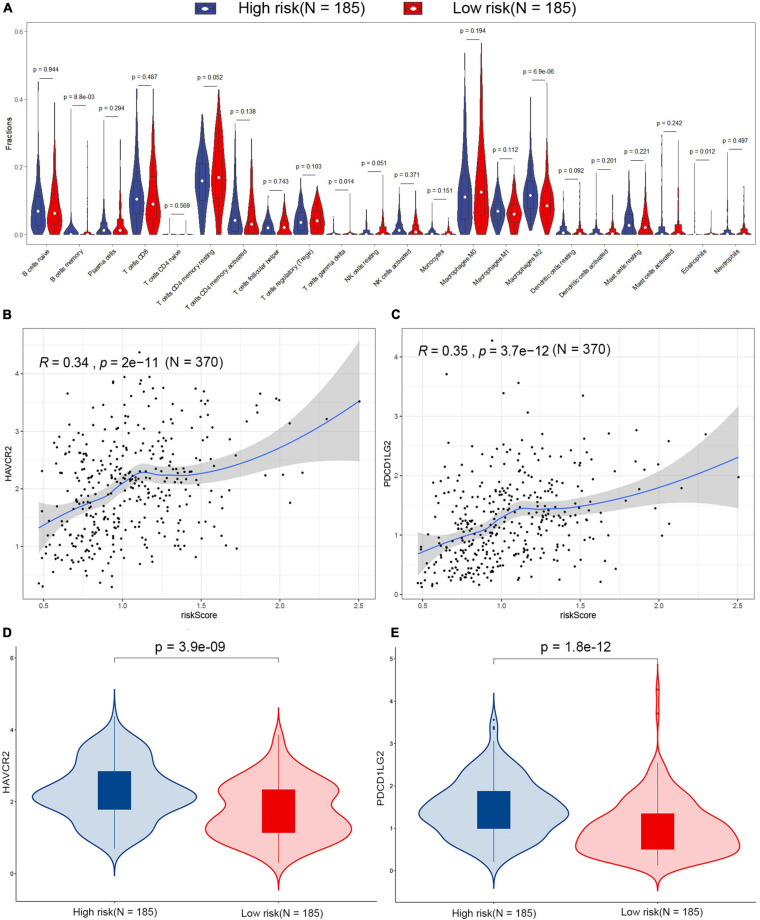
Estimation of the tumor microenvironment in the TCGA dataset. **(A)** Differences in 22 human immune cell phenotypes infiltration between the high- and low-risk groups. Correlations between HAVCR2 **(B)**, PDCD1LG2 **(C)**, and risk score in the TCGA dataset. Comparison of the HAVCR2 **(D)** and PDCD1LG2 **(E)** in low- and high-risk patients in the TCGA dataset.

The difference of tumor-infiltrating immune cell composition between the high-risk and low-risk groups was analyzed by CIBERSORTx. The infiltration levels of four immune cells (M2 macrophages, memory B cells, eosinophils, and gamma delta T cells) were significantly different between the two groups in the TCGA dataset (Figure 6A). However, only the infiltration level of M2 macrophages out of these four immune cells showed a significant difference between high-risk and low-risk groups in the GSE15459 dataset (Supplementary Figure 4). M2 macrophages were highly infiltrated in the high-risk group in both TCGA-STAD and GSE15459 datasets. Besides the infiltrating level of immune cells, we analyzed the correlation between the signature and the expression of different immune checkpoints (PD-L1, PD-1, CTLA-4, TIGIT, LAG-3, TIM-3, PD-L2, IDO-1, IDO-2, ADORA2A, and B7-H4) in the TCGA-STAD dataset (Supplementary Figure 5; Fang et al., 2020; Melaiu et al., 2020). The results showed that the expression of *TIM-3* (also known as *HAVCR2*) and *PD-L2* (also known as *PDCD1LG2*) were positively correlated with risk score, which is more highly expressed in high-risk patients (Figures 6B–E).

### Gene Set Enrichment Analysis of the High-Risk Group

**FIGURE 7:**
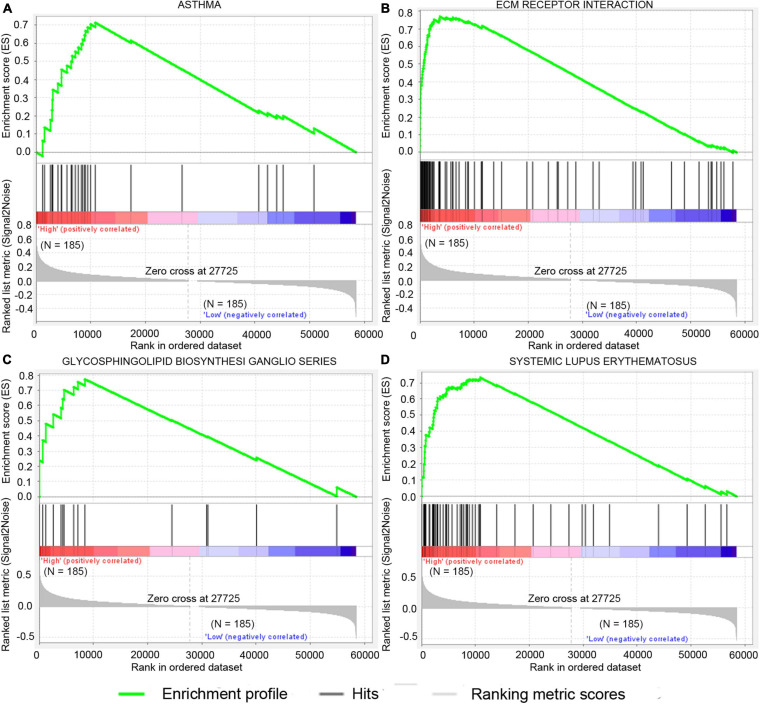
Significantly enriched signal pathway in patients with high risk compared with patients with low risk in the TCGA dataset. The significantly enriched KEGG pathways include asthma **(A)**, ecm receptor interaction **(B)**, glycosphingolipid biosynthesis ganglio series **(C)**, and systemic lupus erythematosus **(D)**.

GSEA analysis was performed to explore potential signaling pathways associated with the high-risk group based on the two-gene signature-derived risk score. The cutoff value was set at FDR *q*-value < 0.05 and |NES| > 0.7. Results showed that four gene sets, namely, asthma, extracellular matrix (ECM)–receptor interaction, systemic lupus erythematosus, and glycosphingolipid biosynthesis-ganglio series, were enriched in the high-risk patients in the TCGA dataset (Figure 7). ECM–receptor interaction was one of the most enriched gene sets in the GSE15459 dataset (Supplementary Table 2).

### Verification of LRFN4 and CTHRC1 Expression in STAD by qRT-PCR

**FIGURE 8:**
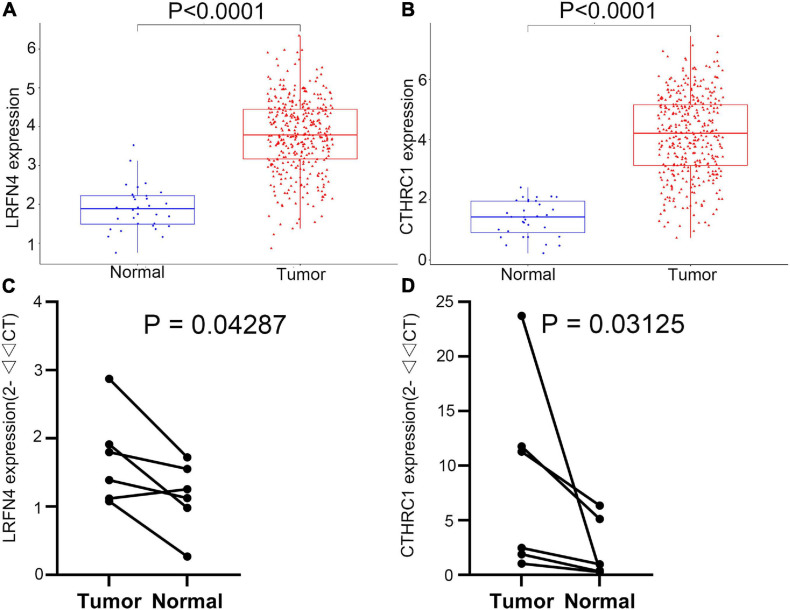
Verification of the expression of *LRFN4* and *CTHRC1*. Expression of *LRFN4* **(A)** and *CTHRC1* **(B)** in the TCGA dataset. qRT-PCR validation of the expression of *LRFN4* **(C)** and *CTHRC1* **(D)**.

The *LRFN4* and *CTHRC1* expression data at the mRNA level were obtained from 407 tissues (including 375 STAD tissues and 32 adjacent normal tissues) in the TCGA database. The plot shows the mRNA expression profiles of *LRFN4* and *CTHRC1* in STAD tissues and adjacent normal tissues. As shown in Figures 8A,B, the expression of *LRFN4* and *CTHRC1* was significantly upregulated in GC tissues compared to that in adjacent normal tissues (*p* < 0.0001). To verify the difference in *LRFN4* and *CTHRC1* expression in the TCGA database, qRT-PCR was used to evaluate the mRNA expression level of *LRFN4* and *CTHRC1*, which showed that their expressions in STAD were both significantly higher than that in adjacent normal tissues (*p* < 0.05, Figures 8C,D).

## Discussion

In the present study, 44 DEGs were commonly identified between STAD and healthy samples from three datasets. To clarify the functions of DEGs, we further performed functional enrichment analysis. The proteins translated by DEGs were mainly located in extracellular regions and these genes were primarily implicated in tumor-related biological processes such as cell adhesion, wound healing, and extracellular matrix organization (Yu et al., 2019; Alfarsi et al., 2020; Liao et al., 2020).

Then, five candidate genes (*DPT*, *ECT2*, *COL5A2*, *CTHRC1*, and *LRFN4*) that were closely related to the survival rate of STAD patients were identified by analyzing the total survival information from STAD patients in the TCGA program. Based on the results of univariate Cox regression and stepwise multivariate Cox regression in the TCGA-STAD dataset, expression of *CTHRC1* and *LRFN4* was applied for construction of risk score and prognostic signature. Kaplan–Meier curves showed that the high-risk group had an obviously poorer overall survival compared to the low-risk group in the three groups. In addition, multivariate Cox regression analyses indicated that the risk score was an independent predictor of survival in TCGA datasets, after adjustment of age, sex, and tumor stage. Risk scores were similar between early (I and II) and advanced (III and IV) stages, but showed significant changes between early stage and normal, which suggested the great potential of the two-gene signature in early diagnosis in patients of STAD. Expression verification analysis of *LRFN4* and *CTHRC1* was performed based on the TCGA database. At the same time, the expression levels of these two genes were verified by qRT-PCR using specimens from our recruited patients.

*CTHRC1* played a role in the cellular response to arterial injury through involvement in vascular remodeling (Pyagay et al., 2005). Previous studies showed that *CTHRC1* promoted tumor cell progression and might play a key role in the invasion metastasis of cervical carcinoma, cervical squamous cell carcinoma, and colorectal cancer (Ni et al., 2018; Li N. et al., 2019; Zheng et al., 2019). In addition, *CTHRC1* promoted M2-like macrophage recruitment and myometrial invasion in endometrial carcinoma by the integrin-Akt signaling pathway, which indicated that *CTHRC1* might be a biomarker for tumor immunotherapy (Li L. Y. et al., 2019).

*LRFN4* encodes leucine-rich repeat and fibronectin type III domain-containing 4, belonging to the superfamily of leucine-rich repeat-containing adhesion molecules (Nam et al., 2011). In a previous study, the expression level of *LRFN4* was high in tumor cells (Liu et al., 2019), which was consistent with present results. However, the high-level expression of *LRFN4* was associated with poor prognosis, which was contradicted with our findings. Because of the unknown relationship between *LRFN4* and cancer, the correlations between *LRFN4* and cancer need to be verified.

Next, immune cell infiltration and GSEA analysis showed that four immune cells and four gene sets have differences between the high-risk and the low-risk groups, but only M2 macrophages and ECM–receptor interaction were confirmed in the GSE15459 dataset. Previous studies revealed that M2 macrophages predominated in human cancers and actively stimulated tumor growth (Mills et al., 2016). ECM is a complex macromolecular network composed of a variety of proteoglycan, fibrin, and stromal cell-associated proteins (Hynes, 2009; Mouw et al., 2014). In addition, the ECM component has a regulatory effect on macrophage polarization (Yunna et al., 2020). Therefore, the poor prognosis of patients in the high-risk group may be due to the effect of M2 macrophages and ECM–receptor interaction.

Immune checkpoint agents had antitumor properties through reverse tumor immunosuppressive effects (Long et al., 2017). The correlation between the signature and the level of different immune checkpoint proteins was also investigated. The high-risk patients with STAD generally had higher expressions of *TIM-3* and *PD-L2*. The positivity of PD-L2 significantly indicated clinical response to pembrolizumab on combined tumor, while stromal immune cells’ T cell immunoglobulin mucin-3 (Tim-3) antibodies have curative effects in laboratory-scale studies in several tumors (Yearley et al., 2017; Liu et al., 2018). Therefore, patients with STAD at high risk may benefit more from immunotherapy. These results suggest that the poor prognosis of patients with STAD at high risk is due to lower immunoreactivity and higher immunosuppression in the tumor microenvironment, which promote the growth, progression, invasion, and metastasis of the tumor.

Bioinformatic analysis played an important role in the potential biomarkers’ discovery for the diagnosis and treatment of stomach-related cancer. A 53-gene signature-derived risk score module was demonstrated to predict prognosis in gastric cancer and a nine-gene signature was identified to predict gastric cancer prognosis (Wang et al., 2016; Wu et al., 2020). However, the complexity of the model decreased its robustness and limited the application of these STAD prognosis signatures on broad datasets. In the present study, a novel two-gene signature was identified, which had simpler structure and stronger early prognostic capacity.

However, there are still some limitations for the signature: (a) The prognostic signature is based on the expression of genes in the tissue, while the expression of genes and proteins in the blood might be more convenient for the clinical application. (b) The prognostic signature has only been verified on two datasets, and needs to be verified on more datasets. (c) The prognostic effect of *CTHRC1* and *LRFN4* had been confirmed, but their specific role in STAD is still unknown. It will be addressed in a future study.

## Conclusion

In conclusion, we constructed a new predictive signature of mRNA prognosis through mRNA expression profiling. The signature contained expression of two genes, which were verified by qRT-PCR analysis using specimens from recruited patients in our hospital. In addition, the fidelity of this signature was evaluated by another independent dataset. Furthermore, we analyzed the possible causes of the difference in prognosis from the perspective of the immune microenvironment. The present study investigated potential biomarkers of STAD based on *in silico* analysis, which laid the foundation for further studies toward understanding of STAD pathogenesis and clinical treatment focusing on prognosis of STAD.

## Data Availability Statement

The original contributions presented in the study are included in the article/Supplementary Material, further inquiries can be directed to the corresponding author/s.

## Ethics Statement

The studies involving human participants were reviewed and approved by the Ethics Committee of The First Affiliated Hospital of Zhejiang University. The patients/participants provided their written informed consent to participate in this study.

## Author Contributions

SH, WZ, and WY conceived and designed the study. SH, QG, RZ, HX, YL, and ZS analyzed the data. SH, WZ, QG, and ZZ wrote the manuscript. WY and CC collected the patient samples. SH, HH, and YL performed the experiments. QH, XH, and JT were involved in project management. XH and JT supervised the study. All authors read and approved the final manuscript.

## Conflict of Interest

WZ was employed by company Changshu Qiushi Technology Co., Ltd. The remaining authors declare that the research was conducted in the absence of any commercial or financial relationships that could be construed as a potential conflict of interest.

## Publisher’s Note

All claims expressed in this article are solely those of the authors and do not necessarily represent those of their affiliated organizations, or those of the publisher, the editors and the reviewers. Any product that may be evaluated in this article, or claim that may be made by its manufacturer, is not guaranteed or endorsed by the publisher.

## Footnotes

1
https://www.ncbi.nlm.nih.gov/geo/

2
https://david.ncifcrf.gov/

3
https://portal.gdc.cancer.gov/

4
http://cibersortx.stanford.edu/

5
http://software.broadinstitute.org/gsea

## Supplementary Material

Supplementary Figure 1 The distribution of adjusted *p*-value in GSE118916 **(A)**, GSE1103236 **(B)**, and GSE13861 **(C)**.

Supplementary Figure 2 The prognostic value of 39 genes in the overall survival of Stomach adenocarcinoma (STAD) patients.

Supplementary Figure 3 Estimation of the performance of 9-gene **(A)** and 53-gene **(B)** signature.

Supplementary Figure 4 Differences in 22 human immune cell phenotypes infiltration between the high- and low-risk groups in GSE15459.

Supplementary Figure 5 Correlations between immune checkpoints and risk score in the TCGA dataset.

Supplementary Table 1 44 DEGs along with *p*-value and log2 fold change in the four datasets.

Supplementary Table 2 Significantly enriched signal pathway in patients with high-risk compared with patients with low-risk in GSE15459.

Supplementary Table 3 Survival analysis of 9 genes (Wu et al., 2020) in TCGA-STAD and GSE15459.

Supplementary Table 4 Survival analysis of signatures containing different genes. Due to too many combinations, each signature chooses the three combinations with the smallest average *p*-value.
