# Supplementary figures and images for: A Prognostic Signature Constructed by CTHRC1 and LRFN4 in Stomach Adenocarcinoma
Source: Front Genet. 2021 Aug 26;12:646818. doi: 10.3389/fgene.2021.646818 (PMC8427509; doi:10.3389/fgene.2021.646818)

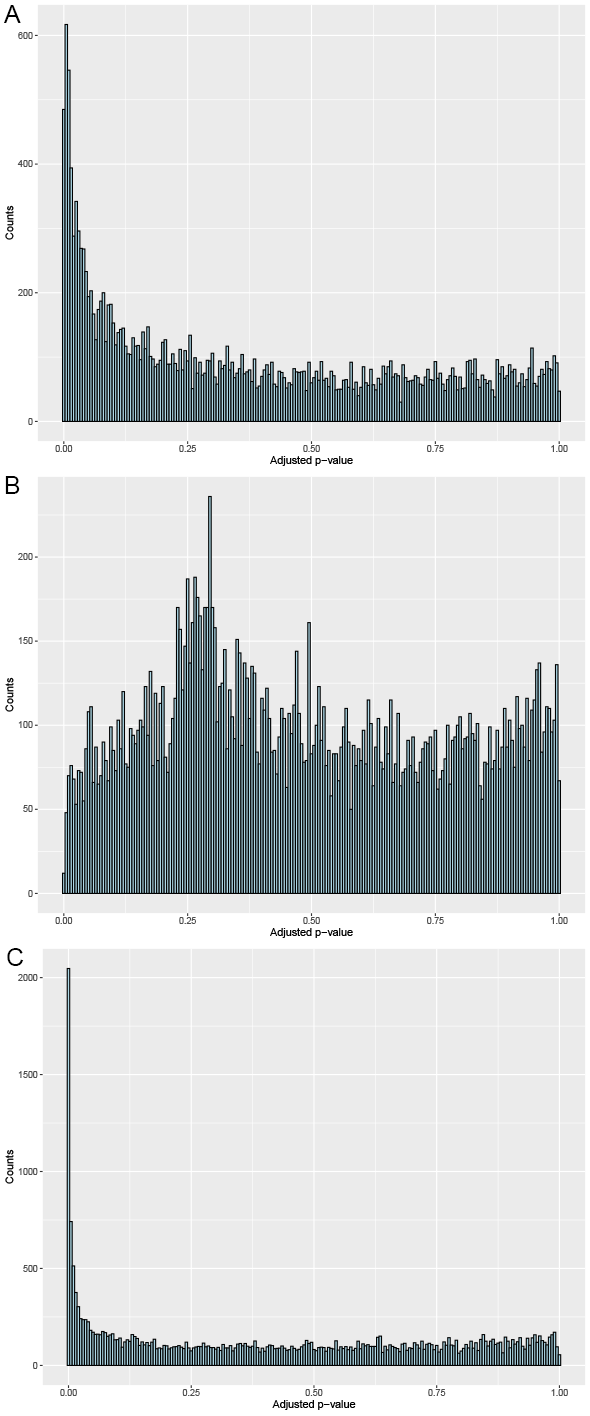

Supplement: Supplementary Figure 1 — The distribution of adjusted p-value in GSE118916 (A), GSE1103236 (B), and GSE13861 (C). [file Image_1.TIF]

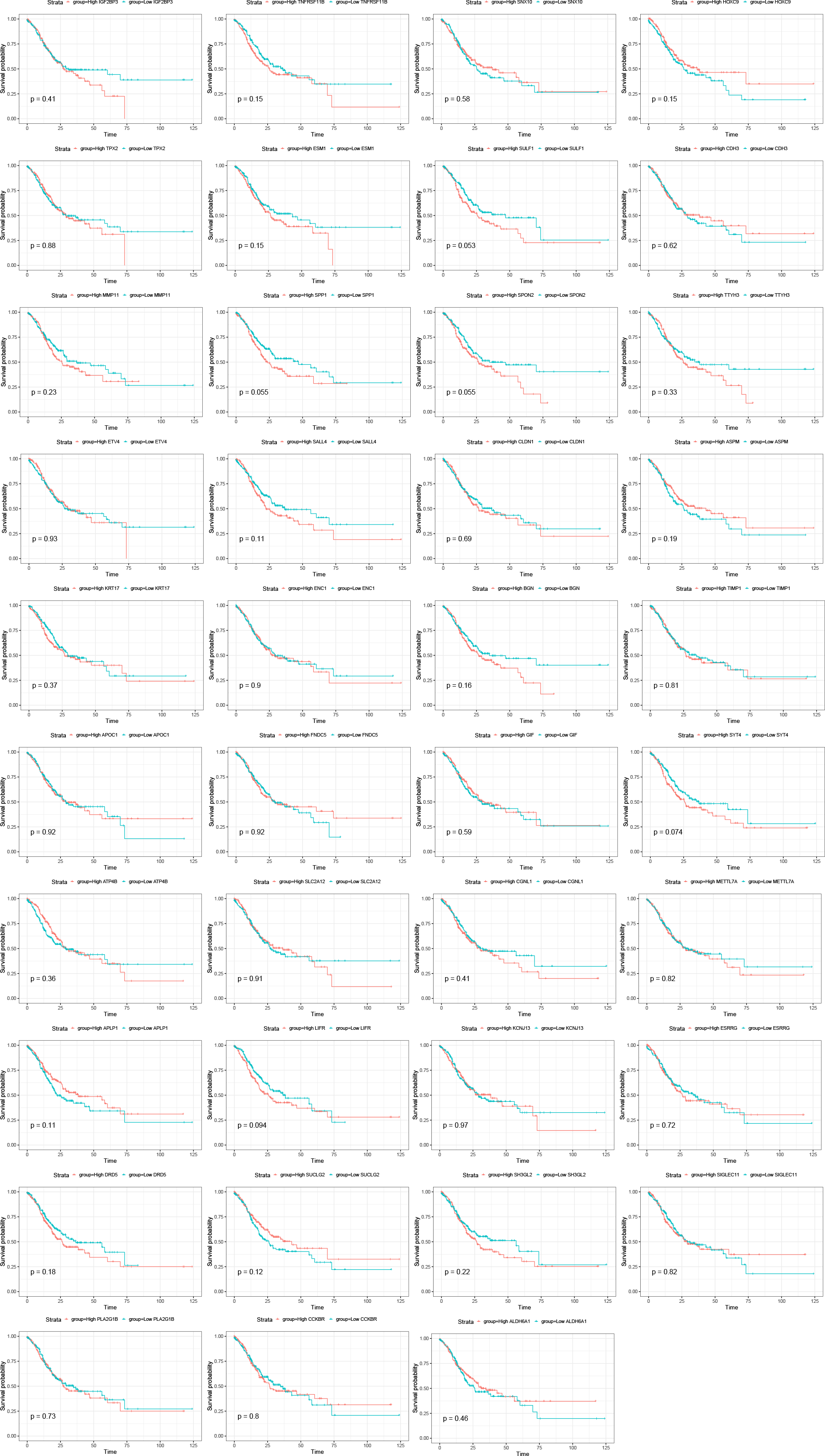

Supplement: Supplementary Figure 2 — The prognostic value of 39 genes in the overall survival of Stomach adenocarcinoma (STAD) patients. [file Image_2.TIF]

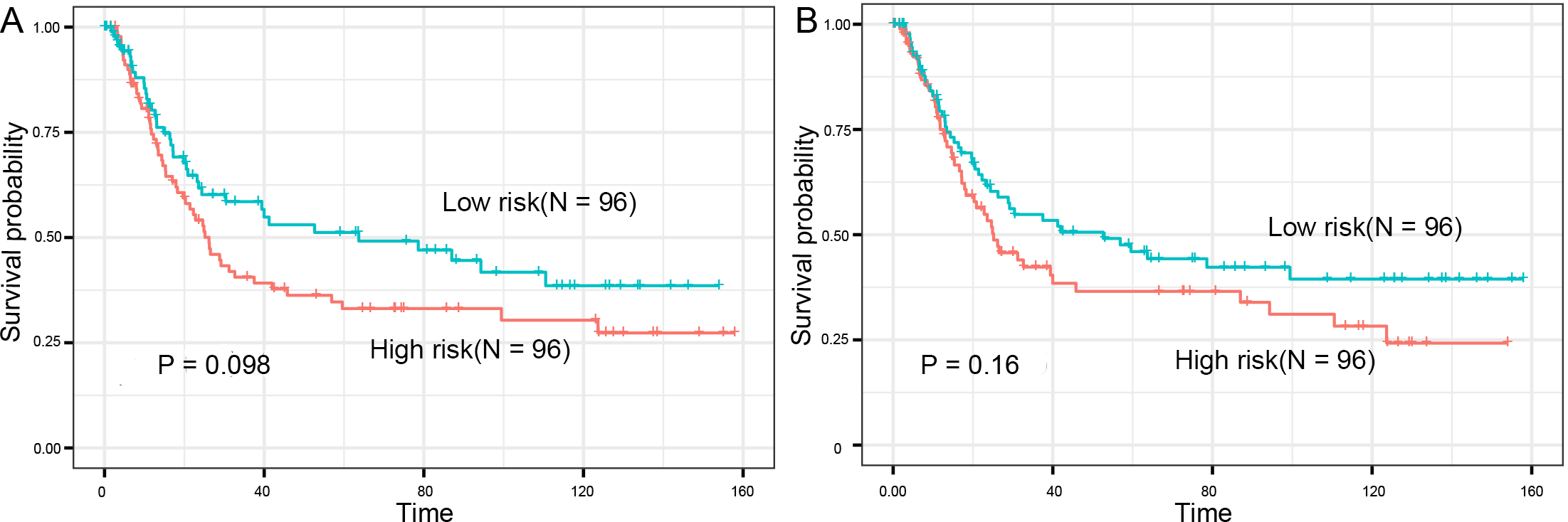

Supplement: Supplementary Figure 3 — Estimation of the performance of 9-gene (A) and 53-gene (B) signature. [file Image_3.TIF]

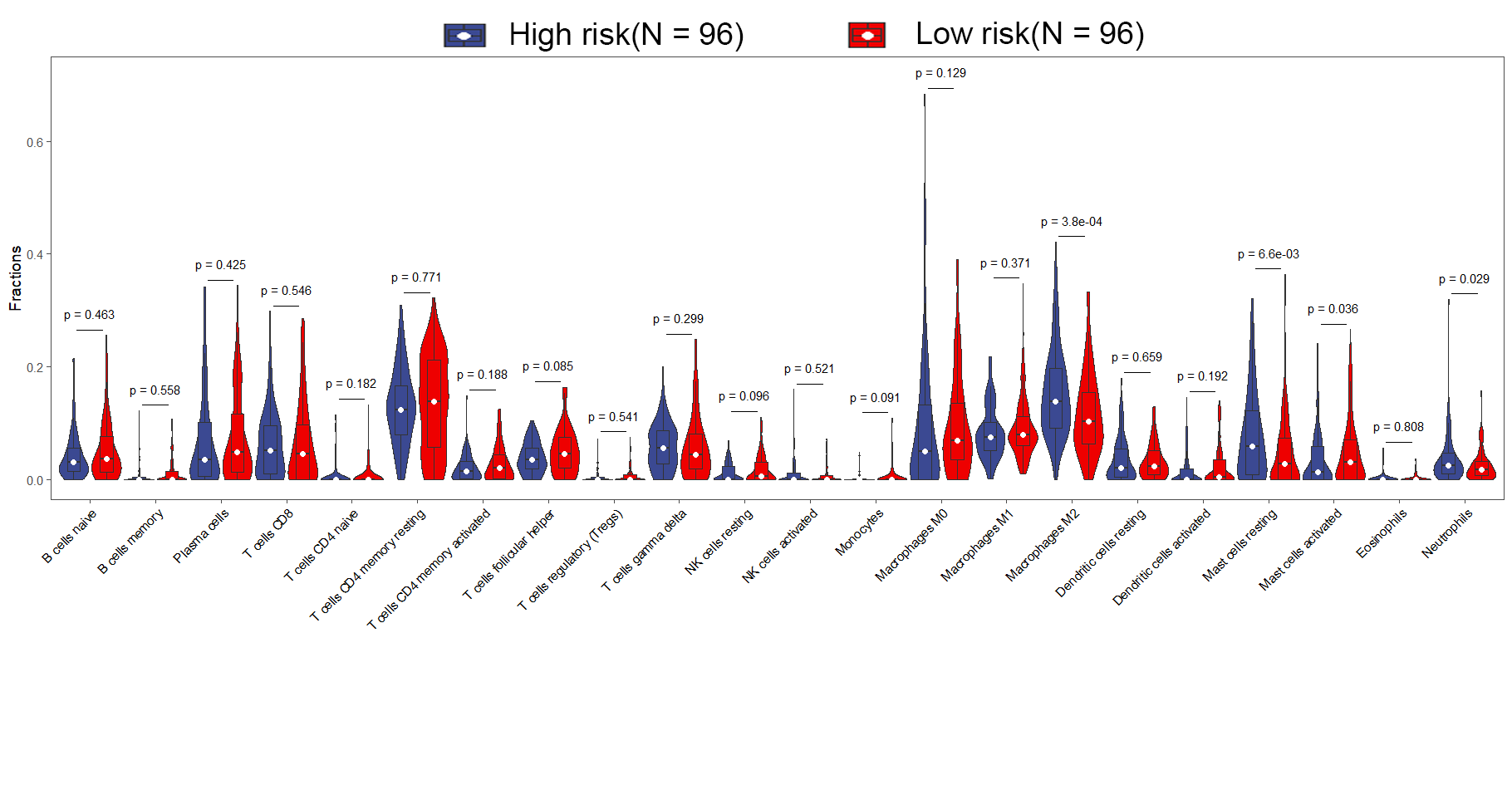

Supplement: Supplementary Figure 4 — Differences in 22 human immune cell phenotypes infiltration between the high- and low-risk groups in GSE15459. [file Image_4.TIF]

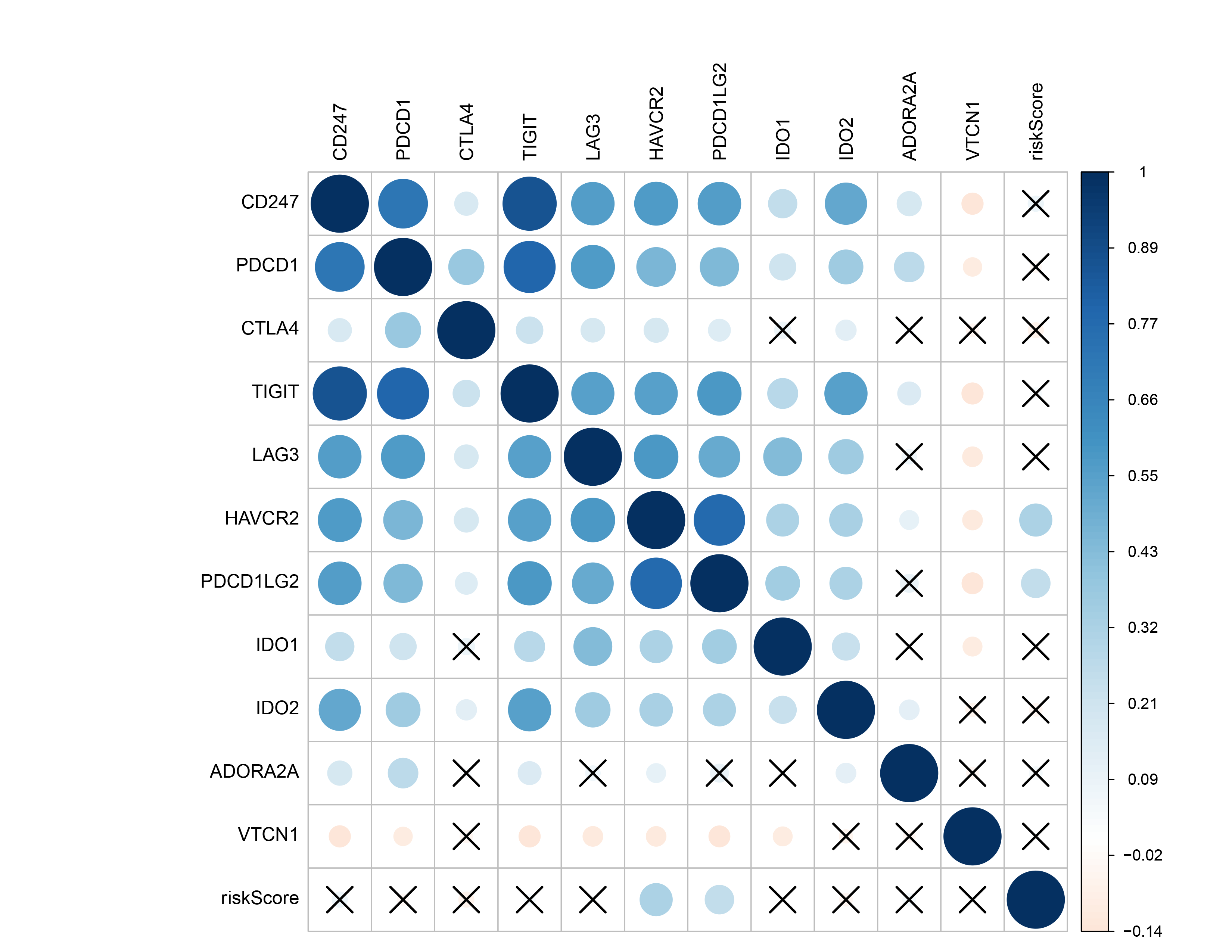

Supplement: Supplementary Figure 5 — Correlations between immune checkpoints and risk score in the TCGA dataset. [file Image_5.TIF]
