# Supplementary material for: A Prognostic Signature Constructed by CTHRC1 and LRFN4 in Stomach Adenocarcinoma
Source: Front Genet. 2021 Aug 26;12:646818. doi: 10.3389/fgene.2021.646818 (PMC8427509; doi:10.3389/fgene.2021.646818)
Supplement: Supplementary Table 1 — 44 DEGs along with p-value and log2 fold change in the four datasets. [file Table_1.DOCX]

| Gene Symbol | HR | HR.95L | HR.95H | P-value |
| --- | --- | --- | --- | --- |
| *LRFN4* | 0.825131 | 0.696212 | 0.977922 | 0.026587 |
| *COL5A2* | 1.231445 | 1.05985 | 1.430822 | 0.006544 |
| *CTHRC1* | 1.205681 | 1.065017 | 1.364924 | 0.003125 |
| *ECT2* | 0.900176 | 0.766669 | 1.056933 | 0.199163 |
| *DPT* | 1.09165 | 0.977869 | 1.21867 | 0.118414 |
